# Investigation of the influence of xanthan on mozzarella cheese characteristics focusing on its antimicrobial effect

**DOI:** 10.5455/javar.2023.j700

**Published:** 2023-09-30

**Authors:** Ayah B. Abdel-Salam, Mena Saad, Rania F. Ahmed, Neveen S. M. Soliman

**Affiliations:** 1 Food Hygiene and Control Department, Faculty of Veterinary Medicine, Cairo University, Giza, Egypt; 2 Agricultural Microbiology Department, Faculty of Agriculture, Ain Shams University, Cairo, Egypt

**Keywords:** Mozzarella cheese, xanthan, chemical parameters, *E. coli* O157, *S. aureus*

## Abstract

**Objective:**

This study was designed to show the effect of adding different levels of microbial (lab-produced) and commercial xanthan (CX) for 30 days on the sensory, chemical, and microbiological parameters of mozzarella cheese (MC).

**Materials and Methods:**

The production of xanthan was done in Garcia–Ochoa’s medium. The sensory evaluation of the examined MC was achieved through a tabulated scorecard*. *The Gerber method was used for the determination of MC fat%. The mean counts of staphylococci [colony forming unit (CFU)/gm], coliforms (most probable number/gm), fungi (CFU/gm), and mesophilic bacteria (CFU/gm) were estimated in different fortified cheeses. Also, mean counts of *Escherichia coli *O157 and *Staphylococcus aureus* in artificially contaminated MC were determined.

**Results:**

The microbial xanthan (MX) had a significant (*p* < 0.05) effect on the sensory parameters of the examined samples with its concentration (0.0007%) after 20 days of storage. The MX (0.0005%) and CX (0.0002%) had a significant effect on moisture, fat in dry matter, and protein percentage of MC throughout the storage period. The high meltability degree of MC was observed in samples with both types of xanthan (0.0002%) at the end of storage.

**Conclusion:**

Both types of xanthan at all concentrations had a significant reducing effect on *E. coli *O157 and *S. aureus* in all samples from 10 to 30 days of storage. Xanthan has accepted attentiveness and offers beneficial and safe characteristics that improve its adaptability in MC. In the Middle East, this survival trial of *E. coli *O157 and *S. aureus* in the MC supplemented by xanthan is considered a scarce exploratory investigation.

## Introduction

Mozzarella cheese (MC) is the most prominent component of pizza. Production of MC has continually elevated because of requirements in pizza manufacturing. Its good, temperate flavor, engaging melt, and smooth texture extensionality make it excellent for pizza [1]. The cheese is soft, white, and made from cow or buffalo milk, which may be eaten shortly after processing. The particular stretching characteristic of MC is significantly acknowledged in pizza processing, in which it is one of the main components [2].

MC is an excellent source of amino acids, minerals, and vitamins. MC may protect you against gout. Calcium in MC reduces body weight and protects against mammary gland cancer and the development of cardiac diseases. Agreeable MC has a flavor that is almost like fresh milk, with a creamy sensibility. Minimal pH and storage at 5°C provide hindrances to the growth of microorganisms during MC storage. The useful lifetime of marketing MC is 30 days [3], although the cheese usually promotes abnormal flavor and damages its textural uprightness within this period. The undesirable texture during this brief useful lifetime is due to proteolysis. The possibility exists that microorganisms and/or their enzymes, such as *Staphylococcus aureus*, produce enterotoxins in food, or the foods may contain enterotoxigenic *Escherichia coli* that produce intestinal diarrheal toxins [4].

Xanthan is a significant source of soluble nutritional fiber when added to cheeses. It is an official food additive in the European Union based on order number 1333/2008 [5]. Xanthan provides steadiness to the emulsion for extended periods, which is an advantage in food manufacturing. These features resulting from xanthan are maintained during shipping, storage, and utilization [6].

Xanthan from microbial sources is used unharmed as a stabilizer in milk products. The soluble fiber ingredients resort to getting away with milk serum, which leads to less cheese retention for an ingredient. Thus, to produce MC with a proper fiber supply, techniques have to be used to obtain the maximum reservation in cheese. However, there is no investigation into the exploitation of lab-produced (microbial) and commercial xanthan (CX) in MC*.* Xanthan significantly increased the fiber content of MC. Since milk does not have enough fiber content, the addition of xanthan to soft cheese has an enhancement impact on its compositional quality [7].

Cell growth, ideal state, and fermentation of xanthan may be variable. The speed, volume, and quality of xanthan produced differ with medium ingredients and ecological factors. Response surface methodology (RSM) is a statistically efficient appliance for an ideal procedure when the unconnected variables have a mutual influence on the in-demand return [8,9]. A preferable model in RSM is the central composite design (CCD), which is malleable and efficient in supplying enough data on the variables, influences, and overall trial mistakes, even with a smaller number [10].

The survivability of different types of microorganisms during MC storage at 5°C has yet to be described. MC has low salt and a shorter useful life. This cheese presents a paradigm to understand the chemical state in soft cheeses that affects microorganism survival with and without xanthan added, thereby changing its quality and safety throughout the storage period [11]. This study is the first, especially in the Middle East, to test whether *E. coli *O157 and *S. aureus* can survive in this newly developed MC enriched with various types and concentrations of xanthan.

Therefore, this study aimed to optimize xanthan production by detecting the most significant factors by Plackett–Burman (PB) design, which was selected for more optimization using RSM through CCD. Also, the objective of this study was to investigate the feasibility of incorporating lab-produced and CX in buffalo MC on the sensory and chemical properties of MC, focusing on the degree of antimicrobial effect of both types of xanthan powder at 0.0002%, 0.0005%, and 0.0007% concentrations during the 30-day storage of MC in a refrigerator (5°C).

## Materials and Methods 

### Techniques for xanthan production in the laboratory

The American Culture Collection in Rockville, MD, is the source of* Xanthomonas campestris* ATCC 13951. The strain was kept at 4°C on the yeast malt (YM) agar slant and was subcultured every 14 days. Propagation was done in YM medium and as a stock culture of *X. campestris* ATCC 13951. Xanthan production was achieved in Garcia–Ochoa’s medium [12,13].

Ten milliliters of samples were withdrawn from the culture every day to determine the growth optical density (OD) and culture viscosity by rotational viscometer, Cole Parmer^®^, by spindle number 5 at 0.6 revolution per minute (RPM). Culture pH was obtained by a waterproof pH tester, AD11 (Adwa^®^, Szeged, Hungary). Xanthan in culture fluid was precipitated, purified, and calculated as a dry weight based on the technique of Sidkey et al. [13].

### Statistical experimental designs for xanthan production optimization 

Monitoring of the most significant factors by the PB pattern using software number 7.0 (Stat-Ease, Inc., Minneapolis, MN) was utilized to assess the proportional value of nutritional and physical items for xanthan processing by the investigated strain. Four nutritional items—sucrose as a carbon source, NH_4_NO_3_ as a nitrogen source, KH_2_PO_4_, and citric acid—and five ecological items (temperature, size of the inoculum, shaking rate, pH, and keeping period) with two dummy items, which give a realistic assessment of the error, were analyzed in 12 (*n* + 1) trials for the investigated strain. Trials were achieved in double, and the mean value was utilized as the design response. Each item was exemplified at two levels (high+, low−) based on Ghashghaei et al. [9].

A CCD was embraced to optimize the major variable after monitoring the significant items for xanthan production by investigating strain using a Placket–Burman design. The three chosen independent items were investigated at three various levels (−1, 0, and +1), and sets of twenty trials (a batch experiment) were achieved by *X. campestris *(three items).

### Chemical parameters for the determination of commercial and produced xanthan

Organic carbon was determined based on Zakeri et al. [14]. Total nitrogen was calculated through the Kjeldahl method, as mentioned by Saez-Plaza et al. [15]. Ash content was analyzed by Harris and Marshall [16].

### Used materials and MC processing 

Buffalo milk for the survival experiment and for other types of examination were obtained from the Faculty of Agriculture, Cairo University Farm, Egypt. An analysis of the gross composition of milk was achieved based on FSSAI [17]. Microbial rennet (Reniplus^®^, 2,000 IMCU/gm) was obtained from Proquiga Company, Spain. Edible-grade salt (sodium chloride) produced by The Company for Salts and Minerals in Egypt was used. Calcium chloride anhydrous was obtained from the Merck Company. The CX (Batch number 210520 with a certificate of quality control testing, ISO 22000: 2018, ISO 9001: 2015) was obtained from Ebos Biotech Co. (Docklands, Australia). Ten milliliters of milk were prepared, and the viscosity was measured using a rotational viscometer using spindle number 5 at 0.6 RPM based on Kumbar and Nedomova [18].

Concentrated lyophilized mixed culture containing *Streptococcus thermophilus *and *Lactobacillus* *delbrueckii subsp*. *bulgaricus*, 1:1, obtained from the Microbiological Resources Center (Egypt) was prepared according to manufacturer directions; rennet, calcium chloride, and salt were added at the ratios of 0.1%, 0.1%, 0.2%, and 2.0%, respectively. The mixture was stirred well and set for 2 h. The cheese, curd after drainage of the whey, was processed in a water bath (GFL, Germany) at 70°C until it gave a desirable MC within 10 min and stored in plastic containers. The containers were tightly closed and stored in the refrigerator (6°C ± 1°C) for 30 days. Also, the purchased commercial MC was kept in the same conditions. 

### Sensory evaluation of MC samples

Each MC sample was numbered and offered to three well-trained panelists when fresh and after 10, 20, and 30 days of storage for flavor (50), body and texture (35), and appearance (15) evaluation [19]. 

### Determination of meltability of MC samples

Five grams of cheese, 2.5 cm in diameter and 0.5 cm thick, were heated in a hot air oven (ECO CELL, Monroe, WA) at 280°C for 4 min. The elevated melted portions (cm) were evaluated three times with graph paper. MC meltability was indicated by the ratio of the melted portion to the original portion [20].

### Chemical parameter examination of MC samples 

Samples of MC were measured for fat, ash, and moisture percentages based on FSSAI [17]. The determination of pH values for cheese samples was achieved using the waterproof pH tester AD11 (Adwa^®^, Szeged, Hungary). The analysis of total protein (TP%) was done using the formol titration method, according to Mendi et al. [21]. The analysis of all these parameters was done three times, and the mean values were calculated.

### Preparation of MC samples for microbial enumeration 

Under sterile conditions, 11 g of cheese sample were switched into 99 ml of diluent containing 2.0% sodium citrate (Sigma–Aldrich) for making the homogenate of cheese. The first dilution was mixed for 10 sec using Stomacher Seward 400 (Worthing, UK) to gain a 1/10 dilution. Then, 1 ml of the first dilution was switched into 9 ml of diluents to gain decimal serial dilutions based on ISO [22], and 0.1 ml of these dilutions were spread on plates of agar. The examination was achieved three times, and counts of staphylococci [colony forming unit (CFU)/gm], coliforms [most probable number (MPN)/gm], yeast and mold (cells/gm), and mesophilic bacteria (CFU/gm) were estimated based on ISO [22].

### Study of E. coli O157 and S. aureus survival in the examined MC

*Escherichia coli *O157 and *S. aureus* strains were used. The *S. aureus* strain was ATCC43300, and the *E. coli *O157 strain was isolated from the examined and surveyed cheeses and molecularly identified by polymerase chain reaction (PCR) technique. Each bacterium was grown on 5 ml of brain heart broth (M210, Himedia) at 37°C for 24 h. At 3500 RPM for 10 min (Thermo-Fisher, USA), the strain was centrifuged. The pellet was washed with peptone water (CM0009: Oxoid) in triplicates. Cells were re-suspended in the same diluent, and the finished concentration was achieved at 10^8^ CFU/ml. Decimal dilutions were done and applied in the inoculation of MC [11].

MC was processed from heated milk and cooled as described by Kiiru et al. [23]. Milk was divided into seven portions: milk with 0.0002%, 0.0005%, and 0.0007% microbial xanthan (MX); milk with 0.0002%, 0.0005%, and 0.0007% CX; and milk without xanthan. Inoculation of milk, which was verified free from *E. coli* and *S. aureus* by PCR, with approximately 7.3 and 6.47 log CFU of *E. coli *O157 and *S. aureus*, respectively, for each type of MC with or without xanthan at each concentration

The portion of cheese samples was inoculated with bacterial strains for testing the survival of *E. coli *O157 and *S. aureus* during 30 days of storage in the refrigerator (5°C). Trials were achieved in triplicate, and means were documented. The carefully chosen percentages of lab-produced and CX were applied according to the preliminary preference test by the panelists.

### Statistical analysis of data

The one-way analysis of variance (ANOVA) was used to estimate the *p*-value (*p *< 0.05) for significant differences between mean values of the measured parameters for different concentrations and types of xanthan-fortified samples using *post-hoc* Tukey HSD through SPSS version 25.

## Results and Discussion

**Figure 1.**
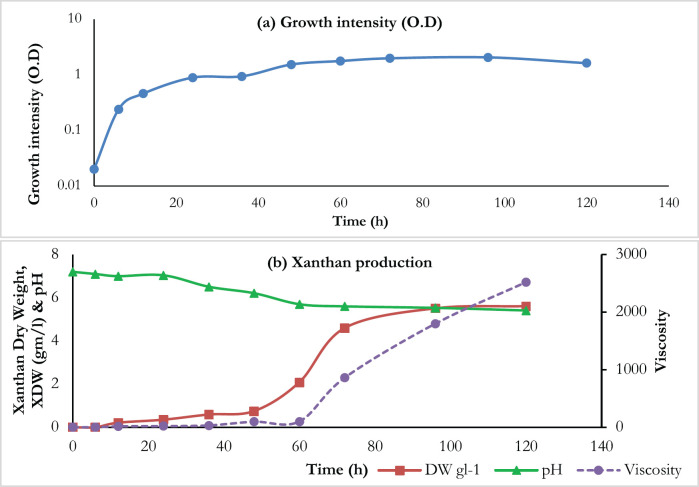
(A) Demonstration the culture growth intensity of X. campestris ATCC 13951 during a 120 h fermentation period in Gracia–Ochoa’s medium. (B) Xanthan production by X. campestris ATCC 13951 in Garcia–Ochoa’s medium during a 120 h incubation period at 28°C using shake flasks as a batch culture.

Figure 1 demonstrates the culture viscosity, OD, and xanthan dry weight of *X. campestris *ATCC 13951 during the 5-day fermentation period in Gracia–Ochoa’s medium. The result in Figure 1a revealed that the investigated strain grew exponentially through the first 12 h of fermentation; thereafter, the growth rate decreased gradually during 12–48 h to be very steady (stationary stage) during the third and fourth days. Then, the decline phase began on the last day of incubation. A slight increase in culture viscosity was observed during the first 12–48 h of the fermentation period. While the speed of elevation was greater during the stationary stage to reach the highest value after 5 days, 2,521 centipoise (CP), and maximum production was 5.61 gm/l (Fig. 1b), a reduction in pH value was noticed during the fermentation period to reach the minimum value (5.41) at the end. These results agreed with those obtained by Sidkey et al. [13]; they noticed the biosynthesis of xanthan followed cell growth from the start of the exponential stage and persisted into the stationary stage.

A statistical experiment of fermentation conditions optimization, PB is an efficient way to monitor the physical and nutritional agents among several process items that influence xanthan production by *X. campestris *ATCC 13951. Nine different variables, including medium constitution (sucrose, ammonium nitrate, KH_2_PO_4_, and citric acid) and culture conditions (pH, incubation period, temperature, size of inoculum, and shaking rate), were selected to achieve the optimization, as displayed in Table 1. The styling has 12 runs with 2 levels for each item at two levels: −1 for a low and +1 for a high.

Results in Table 1 indicated that the production of xanthan ranged from 6.08 to 15.07 gm/l due to the strong influence of interactions between variables on xanthan production. Xanthan production was maximally done at run 12, which consisted of sucrose 40 gm/l, ammonium nitrate 1.71 gm/l, KH_2_PO_4_ 2.86 gm/l, citric acid 4 gm/l, and size of inoculums (5%), keeping period of 5 days, temperature (32°C), pH = 7.2, and speed (400 RPM). The least activity was observed in run one, at 6.08 gm/l. This might be due to low values of both inoculum size (2%) and initial pH used, which eliminate the proper growth of* X. campestris *ATCC 13951 [9, 24].

The Fisher test was used to monitor the impact of independent factors on the answer, and a *p-*value of 0.05 was used to identify results that were significant. Significant for xanthan production was the *F*-value of 8.12. The analyzed outcome of xanthan processes indicates that out of nine independent variables, only three (sucrose, ammonium nitrate, and keeping temperature) significantly influenced xanthan production.

The *R*^2^ was 0.988 for xanthan production by *X. campestris *ATCC13951. This indicates a satisfactory exemplification of process models and a high correlation between the experimental and projected values for the tested strain. It also means that 98.8% of the total variation was explained by the model. In prospect, Ghashghaei et al. [9] established that there are six items that significantly influenced the production of xanthan at two levels: carbon source (30 and 50 gm/l), nitrogen source (1 and 3 gm/l), phosphate (2.5 and 5 gm/l), agitation speed (150 and 250 RPM), size of inoculum (5% and 10%), and pH (6.5 and 7.2).

**Table 1.** PB experimental design matrix, the actual values of xanthan produced by *X. campestris *ATCC 13951, and its statistical ANOVA.

Run number	Variables												Xanthan produced (gm/l)	
	A	B	C	D		E	F	G	H	I	J	K	Actual	Predicted
1	+1	−1	+1	+1		−1	+1	+1	+1	−1	−1	+1	6.08	5.77
2	+1	−1	+1	−1		+1	−1	+1	−1	1	−1	−1	6.5	5.75
3	−1	+1	+1	+1		−1	−1	−1	+1	−1	+1	−1	6.8	6.64
4	+1	−1	+1	+1		+1	−1	−1	+1	1	−1	+1	7.7	7.54
5	−1	−1	+1	+1		+1	−1	+1	−1	−1	+1	+1	7.9	8.16
6	−1	+1	+1	+1		+1	−1	+1	−1	+1	+1	−1	8.0	8.37
7	+1	+1	−1	−1		−1	+1	−1	+1	+1	−1	+1	9.5	9.51
8	+1	+1	+1	−1		−1	+1	−1	+1	+1	−1	−1	11.5	11
9	+1	+1	+1	+1		−1	+1	+1	+1	+1	+1	+1	12.5	12.37
10	−1	+1	−1	+1		+1	−1	−1	−1	+1	−1	−1	13.5	13
11	−1	+1	−1	−1		−1	+1	+1	−1	+1	+1	−1	14.6	14.42
12	+1	+1	+1	+1		+1	+1	+1	+1	+1	−1	+1	15.08	15.05

Variables			Real levels			Coefficient means squares			df		*F*-value			*p*-value (prob. > *F*)
			−1		1	-			-		-			-
Model			-		-	0.045			35		8.12			0.013
A—Sucrose (gm/l)			20		40	3.14E-04			1		129.92			<0.0001
B—NH_4_NO_3_ (gm/l)			1.144		1.71	3.81E-04			1		24.49			0.004
C—KH_2_PO_4_ (gm/l)			2		2.86	6.34E-04			1		0.0521			0.829
D—Citric acid (gm/l)			2.1		4.0	0.081			1		0.1768			0.692
E—Inoculum size (ml)			2		5	1.16E-04			1		5.44			0.067
F—Incubation period (day)			4		5	0.11			1		0.0052			0.945
G—Temperature (°C)			30		32	0.23			1		8.24			0.035
H—Agitation speed (RPM)			150		400	1.75E-05			1		3.88			0.106
I—pH			6.9		7.2	3.80E-05			1		4.83			0.079
J—Dummy 1			-		-	0.25			1		0.0108			0.921
K—Dummy 2			-		-	0.076			1		4.72			0.082
Standard deviation			-						0.93					
Mean			-						10.22					
*R*^2^ (determination coefficient)			-						0.988					

A–H = nutritional and physical variables, −1 = low level of the variable, +1 = high level of the variable, 0 = medium level of the variable, J and K = dummy variables, df = degree of freedom, significant at 5% level (*p* < 0.05).

The CCD was used to determine the best conditions for maximizing the activity of *X. campestris* ATCC 13951 for xanthan synthesis, utilizing three factors at three levels, as indicated in Table 2. This was done after screening the components and their interactions. The highest production of xanthan from these trials, along with the existing and predicted values, was recorded at run number 20 (16.22 gm/l), followed by run number 13 (14.5 gm/l). In similar studies, Zakeri et al. [14] found that the RSM is an efficient way to optimize the production of xanthan by *X. campestris*. Shaking speed, carbon origin amount, and temperature were selected as independent factors in the xanthan biopolymer and biomass production. It could be stated that the highest actual value of xanthan production (16.22 gm/l) was assembled for the predicted value by *X. campestris* ATCC 13951 when fermentation media was inoculated with a 5% inoculum size and kept at 32^°^C for 5 days using a rotary shaker at 400 RPM.

Table 3 provides a summary of the *F*-test and ANOVA results for the response surface quadratic model. The model was significant (*p* < 0.0001), and the *F*-value of 22.04 indicated that there was only a 0.01% chance. The *R*^2^ of the model was 0.95, which indicates that 95% of the total differences were demonstrated by the model and that good endorsement was detected between the experimental outcome and the predicted values calculated from the model. From the degrees of significance, the coefficients of the model term, one variable, temperature (C), and intercommunication between two variables (AC, A^2^, B^2^, C^2^, C, and B) have a significant influence on xanthan production. The highest probability (*p*-value) was monitored in the C variable (temperature), followed by intercommunication between *A* (sucrose) and C (temperature) being less than 0.0001 and 0.043, respectively. Xanthan produced by *X. campestris* ATCC 13951 was nearly similar in physical properties (color, water solubility, and viscosity) to CX. Nevertheless, for chemical factors, produced xanthan displayed less carbon content and greater nitrogen and ash content than CX (Table 4); this indicates the economical aspect of the produced xanthan. Ngowatana and Rudisirisak [25] observed that the viscosity, pH, melting point, and infrared spectrum of both produced and CX were similar.

**Table 2.** CCD of independent variables used in RSM studies and xanthan produced (actual and predicted values) by *X. campestris *ATCC 13951*.*

Run number	Variables			Xanthan production (gm/l)		
	A	B	C	Actual		Predicted
1	+1	0	0	13.21		12.19
2	0	−1	0	11.43		11.05
3	0	−1	0	5.25		6.01
4	−1	−1	+1	12		11.08
5	0	0	0	4.75		4.5
6	−1	0	0	10.07		10.00
7	+1	+1	−1	12.44		13
8	+1	−1	+1	8.67		8.51
9	−1	+1	+1	10.09		10.11
10	0	0	0	5.01		4.91
11	0	0	0	4.09		4.0
12	0	0	+1	7.89		7.7
13	+1	−1	−1	15.5		15. 43
14	0	+1	0	9.78		9.66
15	−1	−1	−1	14.5		14.01
16	−1	+1	−1	13.11		13.0
17	−1	+1	+1	5.67		5.5
18	0	0	0	4.91		4.88
19	0	0	0	4.05		4.5
20	0	0	−1	16.22		16. 35
**Variables**	**Symbol**			**Real levels**		
				**−1**	**0**	**+1**
Sucrose (gm/l)	A			40	50	60
NH_4_NO_3_ (gm/l)	B			1.71	2.5	3
Temperature (°C)	C			32	34	36

A–G = Nutritional and physical variables, −1 = low level of the variable, +1 = high level of the variable, 0 = medium level of the variable.

Generally, the cultural states for xanthan production were optimized using the most significant factors, which were detected by the PB design. In addition, the significant items were chosen for higher optimization by RSM through CCD. The predicted value of xanthan concentration (16.35 gm/l) was agreed with the experimental value as a result of the models from CCD. These optimized conditions led to a 2.89-fold increase in the xanthan concentration compared to that obtained in Garcia–Ochoa’s medium containing 2% sucrose and 0.11% ammonium nitrate inoculated at 2% inoculum size and incubated for 5 days at 28°C on a rotary shaker at 200 RPM.

Based on the chemical composition of buffalo milk used for processing MC, the fat%, protein%, lactose%, total solid (TS)%, moisture%, solids not fat (SNF)%, ash%, and pH values were 6.8, 2.79, 3.92, 14.33, 85.67, 7.45, 0.72, and 6.6, respectively (Table 5). The changes in milk viscosity (CP) were recorded in Table 6 as follows: 32, 50, 58, 36, 57, 61, 13, and 20 for milk with MX (0.0002%, 0.0005%, and 0.0007%), milk with CX (0.0002%, 0.0005%, and 0.0007%), raw milk, and pasteurized milk, respectively.

**Table 3.** Statistical ANOVA of CCD design for produced xanthan by *X. campestris *ATCC 13951.

Variables	Xanthan polymer production			
	Coefficient means squares	df	*F*-value	*p*-value (prob. > *F*)
Model	299.04	9	22.04	<0.0001
A—sucrose	0.3574	1	0.2370	0.6369
B—NH_4_NO_3_	10.39	1	6.89	0.0254
C—temperature	82.57	1	54.77	<0.0001
AB	0.9045	1	0.5999	0.4565
AC	8.02	1	5.32	0.0438
BC	0.0351	1	0.0233	0.8817
A^2^	81.56	1	54.10	<0.0001
B^2^	57.48	1	38.12	0.0001
C^2^	95.96	1	63.65	<0.0001
Standard deviation	1.23			
Mean	9.44			
*R* ^2^	0.95			

df = degree of freedom, significant at 5% level (*p* < 0.05).

In Table 7, the highest total sensory score was gained in the examined MCMX 0.0007% at zero and 20 days of storage. The MC acceptability does not significantly differ (*p *> 0.05) at 10 and 30 days of storage with lab-produced xanthan (0.0007%) and CX (0.0005%) in comparison with CMC; this indicates that these concentrations of xanthan could contribute to reaching the same high grading score as commercial MC. In our study, samples with MX (0.0002%), CX (0.0002%), and control samples were slightly loosened and not as elastic and cohesive as the rest of the MC samples. MCCX 0.0005% samples were the best in appearance, taste, smell, chewing, and soft consistency. Faster and poorer yellowish changes, yeast odor, sour taste, and sticky texture were noticed in the fourth week in stored commercial MC samples. MCCX 0.0007% samples showed a bitter taste that began to appear after 21 days, and it persisted and elevated obviously at the end of the fourth week. The cause of fewer firms for cheese containing xanthan was due to the debilitated micelles grid, and the softer body was due to the greasy fat effect, and milk fat hinders firm protein matrix formulation [26].

**Table 4.** Comparison between physicochemical properties of CX and the xanthan produced using shack flasks as a batch culture.

Properties	CX	Lab-produced xanthan
Color	White powder	Off white
Solubility	Soluble in water	
Viscosity	1,500 CP	1,450 CP
Carbon content %	45.0	39.45
Total nitrogen %	0.30	0.32
Ash %	13.0	14.7

**Table 5.** Gross composition of milk used in MC manufacture.

F	6.8
P	2.79
L	3.92
TS	14.33
M	85.67
SNF	7.45
Ash	0.72
pH value	6.6

Values equal percentage, F = fat, P = protein, L = lactose, TS = total solids, M = moisture, SNF = solids not fat.

**Table 6.** Changes in viscosity of milk used in MC manufacture after adding of various concentrations of xanthan.

Sample	Viscosity (CP)
Milk with MX, 0.0002%	32
Milk with MX, 0.0005%	50
Milk with MX, 0.0007%	58
Milk with CX, 0.0002%	36
Milk with CX, 0.0005%	57
Milk with CX, 0.0007%	61
Raw milk without xanthan	13
Pasteurized milk	20

MX = microbial xanthan, CX = commercial xanthan, CP = centipoise.

The addition of xanthan influenced the sensory parameters of MC. The texture rating was the highest in the CX 0.0005% containing MC, with a score of 34, 32, 32, and 30 at 0, 10, 20, and 30 days of storage, respectively. Further addition of CX to 0.0007% negatively affected the texture of the produced MC. Oberg et al. [27] indicated xanthan use of up to 1% to improve the texture of MC as compared to control samples. Furthermore, xanthan has been recorded as an MC texture enhancer, which is considered one of the technological improvement aspects [28].

In comparison to the control samples, the flavor rating was higher in the cheeses that contained lab-produced xanthan (0.0007%) at zero, 20, and 30 days of storage. The flavor of the examined cheeses with lab-produced xanthan (0.0007%) was rated higher than those with CX (0.0002%). This signals a flavor improvement in MC. A decrease in fat has an impact on nonpolar fat features, such as flavor-carrying capacity, according to Sattar et al. [29]. The low fat has an unfavorable color as matched with full-fat MC. Other researchers have reported the ability of xanthan to enclose flavor in edible products, which may explain the withholding of flavors in MC [7].

Accordingly, in sensory evaluation, MC with lab-produced xanthan (0.0007%) was significantly evaluated higher in the total score as compared to the control until day 20 of storage. Lab-produced xanthan 0.0007% MC samples had the same nonsignificantly diverse (*p *> 0.05) grade as the samples without xanthan at day 30 of storage. Overall sensory acceptability was also evaluated as best for samples that contained MX 0.0007% over the course of storage. This signals that MX has significantly improved MC’s attractiveness. The elevated degree of solution pseudo-plasticity brought about by the xanthan’s existence makes the aqueous system an efficient mix, which leads to excellent mouth organoleptic parameters, especially without sliminess [30].

**Table 7.** Grading of the investigated MC at different storage periods.

Treatment Aspect	MCMX, 0.0002%	MCMX, 0.0005%	MCMX, 0.0007%	MCCX, 0.0002%	MCCX, 0.0005%	MCCX, 0.0007%	MC without X	CMC
Storage period (zero-day)								
Flavor (50)	40	43	45	43	40	40	40	45
B and T (35)	25	30	32	28	34	26	30	30
Appearance (15)	10	8	13	11	14	12	12	13
Total (100)	75 ± 0.41^a^	81 ± 0.41^c^	90 ± 0.41^e^	82 ± 0.41^c^	88 ± 0.41^d^	78 ± 0.41^b^	82 ± 0.41^c^	88 ± 0.41^d^
Storage period (10 days)								
Flavor	40	42	45	43	46	46	38	45
B and T	30	30	33	29	32	31	29	34
Appearance	10	12	13	12	14	13	12	13
Total (100)	80 ± 0.47^ a^	84 ± 0.47^ b^	91 ± 0.47^cd^	84 ± 0.47^b^	92 ± 0.47^d^	90 ± 0.47^c^	79 ± 0.47^a^	92 ± 0.47^d^
Storage period (20 days)								
Flavor	41	39	45	42	40	30	42	44
B and T	30	31	33	31	32	30	34	33
Appearance	10	12	14	13	12	13	12	14
Total (100)	81 ± 0.47^b^	82 ± 0.47^b^	92 ± 0.47^f^	86 ± 0.47^d^	84 ± 0.47^c^	73 ± 0.47^a^	88 ± 0.47^e^	91 ± 0.47^f^
Storage period (30 days)								
Flavor	40	39	43	40	39	30	40	41
B and T	29	31	30	28	30	28	33	32
Appearance	10	11	12	13	12	11	12	13
Total (100)	79 ± 0.47^b^	81 ± 0.47^c^	85 ± 0.47^d^	81 ± 0.47^c^	81 ± 0.47^c^	69 ± 0.47^a^	85 ± 0.47^d^	86 ± 0.47^d^

B&T = body and texture, MCMX = mozzarella cheese with microbial xanthan, MCCX = mozzarella cheese with commercial xanthan, MC = mozzarella cheese, X = xanthan, CMC = commercial mozzarella cheese, ± = standard error.

Different superscript letters in the same row indicate significant differences (*p < *0.05).

**Figure 2.**
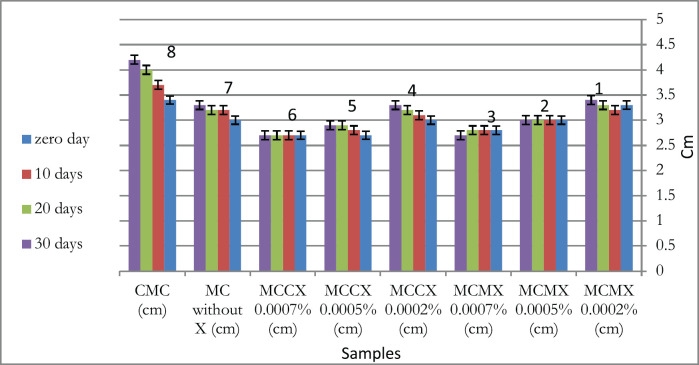
Effect of both types of xanthan on meltability of MC during different storage periods. CMC = commercial mozzarella cheese, cm = centimeter, MC = mozzarella cheese, X = xanthan, MCCX = mozzarella cheese with commercial xanthan, MCMX = mozzarella cheese with microbial xanthan. Illustrated values as mean, error bars indicate ± standard error. For all storage periods, there was a significant difference (*p < *0.05) between control samples and CMC. For zero-day storage; there was a significant difference (*p < *0.05) between 4, 5, 6, and control samples and CMC, and there was a significant difference (*p < *0.05) between 1 and control samples. For 10-day storage; there was a significant difference between 2, 3,4,5,6, and control samples and CMC. For 20-day storage; there was a significant difference between 3, 6, and control samples and CMC. For 30-day storage; there was a significant difference between 3, 5, 6, and control samples and CMC.

Meltability is the cheese’s capacity to flow in an uninterrupted, regular melted mass [31]. The addition of CX at 0.0005% and 0.0007% had a significant influence (*p *< 0.05) on the meltability of MC throughout the storage periods (Figure 2). Thus, xanthan had the possibility of improving MC meltability when added at elevated concentrations. Minimizing fat content in MC decreases the meltability number. Xanthan can improve the functionality of MC fat, according to Oberg et al. [27], who used xanthan in low-fat MC and enhanced the meltability of the processed MC cheese. Thus, xanthan at low percentages can be applied as a fat function substitute with minimal changes in the meltability of MC [29].

Given the degree of MC meltability at zero-day, there was a significant difference between all samples containing xanthan and samples without xanthan except MCMX (0.0005% and 0.0007%) samples. At 10-day storage, there was a significant difference (*p *< 0.05) between all samples containing xanthan and samples without xanthan, except samples MCMX 0.0002%. At 20 days of storage, there was a significant difference between all samples containing xanthan and samples without xanthan, except samples MCMX (0.0002% and 0.0005%) and MCCX (0.0002% and 0.0005%). At 30 days of storage, there was a significant difference between all samples containing xanthan and samples without xanthan, except samples MCMX (0.0002% and 0.0005%) and MCCX (0.0002%). These indicated that lab-produced xanthan (0.0002%) can be added to reach the same high meltability degree as commercial MC throughout the storage period (Figure 2).

Both the amphiphilic and hydrophilic characteristics of xanthan enhance its ability to bind H_2_O and fat in edible products. The reduction of free oil formation in MC containing xanthan can be caused by the emulsifying abilities of xanthan [32].

Xanthan behaves by trapping excess water, which makes lubricity resemble full-fat output. It is a binder of water in milk products [33] that can bind H_2_O in MC, resulting in stretchability (increasing in diameter during melting) and improvement in the final MC quality. The MC stretch properties are influenced by the interconnection between micelles. The more the MC stretches, the more the casein network is joined. Furthermore, if the interactivity between micelles is damaged, the stretchability of MC is reduced. The little interrelation between micelles would also enhance melting and stretchability characteristics, which are spotted at 4% xanthan [2].

Based on chemical aspects of the examined MC samples after adding xanthan throughout storage, for moisture percentage, there was a significant difference between MCMX (0.0005%), MCCX (0.0002% and 0.0007%), and samples without xanthan. While for fat in dry matter percentage, there was a significant difference between all samples containing xanthan and without xanthan until 20 days of storage. For the protein percentage, there was a significant difference between all samples containing xanthan except MCCX (0.0007%) and control samples from 10 days until the end of the storage period. For ash%, there was a significant difference between all samples with xanthan and samples without xanthan from 10 to 30 days of storage. For the pH value, there was a significant difference only between samples MCMX (0.0002%) and others at zero-day, while there was a significant difference between samples with CX (0.0005% and 0.0007%) and samples without xanthan at 10 and 20 days of storage (Table 8).

Xanthan had a slightly elevated water percentage in the final MC. The difference was nonsignificant (*p *> 0.05) in lab-produced xanthan (0.0002% and 0.0007% till day 20 storage) compared to CX. This may be referred to as the efficient formulation of xanthan in large-scale processing plants. Results were obtained without any increase in moisture content when MX was utilized as a fat substitute in low-moisture partial skimmed MC [27].

A further study that supports our study found that adding xanthan in solution form increased the amount of water present [7]. The moisture content values ranged from 39.59% to 44.12% (Table 8), which is classified as low moisture MC [34]. The protein percentage of MC ranged between 23.26% and 25.87%. This was comparable to the [35] investigation. There was a significant reduction in protein percentage except for MCCX 0.0007% samples in comparison with control samples at 10 days of storage, while control samples had the highest protein content, which was not significantly (*p *> 0.05) different from samples MCCX 0.0007% and commercial samples at zero and 10 days of storage. This significant reduction in protein percent may be due to the low protein percent (4.55) of xanthan, as matched with cheese, and the increased water absorption ability of xanthan [7]. The ash variation in MC was due to a variation in the water content of MC. Samples of MC with xanthan show a higher pH; this may refer to the buffering influence of xanthan (pH 7.2) during MC processing and storage [36].

**Table 8.** Changes in chemical aspects of the examined MC samples after adding of xanthan.

Aspect Samples	M	Fat in dry matter	Proteins	Ash	pH value
At zero day					
MCMX, 0.0002%	39.98 ± 0.07^ab^	54.67 ± 0.08^b^	25.32 ± 0.07^a^	4.81 ± 0.08^c^	6.1 ± 0.08^a^
MCMX, 0.0005%	40.23 ± 0.07^c^	54.97 ± 0.08^cd^	25.36 ± 0.07^a^	4.88 ± 0.08^c^	7.5 ± 0.08^c^
MCMX, 0.0007%	40.55 ± 0.07^d^	55.23 ± 0.08^e^	25.42 ± 0.07^a^	4.89 ± 0.08^c^	7.4 ± 0.08^c^
MCCX, 0.0002%	40.11 ± 0.07^bc^	55.12 ± 0.08^de^	25.44 ± 0.07^a^	4.72 ± 0.08^bc^	7.5 ± 0.08^c^
MCCX, 0.0005%	40.54 ± 0.07^d^	55.81 ± 0.08^f^	25.54 ± 0.07^ab^	4.77 ± 0.08^bc^	7.4 ± 0.08^c^
MCCX, 0.0007%	40.98 ± 0.07^e^	56.11 ± 0.08^g^	25.73 ± 0.07^bc^	4.79 ± 0.08^bc^	7.5 ± 0.08^c^
MC without X	39.81 ± 0.07^a^	49.84 ± 0.08^a^	25.80 ± 0.07^bc^	4.51 ± 0.08^ab^	7.4 ± 0.08^c^
CMC	42.13 ± 0.08^f^	54.73 ± 0.08^bc^	25.81 ± 0.09^c^	4.31 ± 0.09^a^	7.1 ± 0.08^b^
At 10 days					
MCMX, 0.0002%	40.32 ± 0.08^a^	54.23 ± 0.07^b^	25.11 ± 0.08^a^	4.66 ± 0.08^c^	7.9 ± 0.08^cd^
MCMX, 0.0005%	40.67 ± 0.08^b^	54.54 ± 0.07^c^	25.21 ± 0.08^ab^	4.61 ± 0.08^c^	8.0 ± 0.08^d^
MCMX, 0.0007%	41.21 ± 0.08^c^	54.83 ± 0.07^d^	25.17 ± 0.08^ab^	4.60 ± 0.08^c^	7.9 ± 0.08^cd^
MCCX, 0.0002%	40.78 ± 0.08^b^	54.99 ± 0.07^d^	25.24 ± 0.08^ab^	4.70 ± 0.08^c^	7.9 ± 0.08^cd^
MCCX, 0.0005%	41.45 ± 0.08^c^	55.63 ± 0.07^e^	25.33 ± 0.08^ab^	4.73 ± 0.08^c^	7.6 ± 0.08^ab^
MCCX, 0.0007%	41.77 ± 0.08^d^	55.89 ± 0.07^e^	25.60 ± 0.08^bc^	4.77 ± 0.08^c^	7.7 ± 0.08^bc^
MC without X	41.40 ± 0.08^c^	48.95 ± 0.07^a^	25.68 ± 0.08^cd^	3.92 ± 0.08^a^	8.0 ± 0.08^d^
CMC	42.54 ± 0.08^e^	54.12 ± 0.08^b^	25.87 ± 0.08^d^	4.28 ± 0.08^b^	7.4 ± 0.08^a^
At 20 days					
MCMX, 0.0002%	41.12 ± 0.07^a^	53.87 ± 0.08^cd^	24.72 ± 0.09^cd^	4.31 ± 0.07^b^	7.8 ± 0.08^c^
MCMX, 0.0005%	41.12 ± 0.07^a^	53.23 ± 0.08^b^	24.21 ± 0.09^b^	4.34 ± 0.07^b^	7.5 ± 0.08^b^
MCMX, 0.0007%	41.89 ± 0.07^c^	53.79 ± 0.08^c^	24.17 ± 0.09^b^	4.32 ± 0.07^b^	7.6 ± 0.08^bc^
MCCX, 0.0002%	41.39 ± 0.07^a^	54.12 ± 0.08^d^	24.24 ± 0.09^b^	4.41 ± 0.07^b^	6.9 ± 0.08^a^
MCCX, 0.0005%	42.79 ± 0.07^b^	54.95 ± 0.08^e^	24.33 ± 0.09^b^	4.44 ± 0.07^b^	7.4 ± 0.08^b^
MCCX, 0.0007%	42.99 ± 0.07^b^	55.32 ± 0.08^f^	24.39 ± 0.09^bc^	4.40 ± 0.07^b^	7.5 ± 0.08^b^
MC without X	41.79 ± 0.07^c^	48.00 ± 0.08^a^	23.77 ± 0.09^a^	3.69 ± 0.07^a^	7.8 ± 0.08^c^
CMC	42.77 ± 0.07^b^	53.23 ± 0.08^b^	24.99 ± 0.09^d^	3.98 ± 0.07^a^	7.6 ± 0.08^bc^
At 30 days					
MCMX, 0.0002%	42.33 ± 0.08^c^	50.78 ± 1.74^ab^	23.89 ± 0.08^d^	4.00 ± 0.07^bc^	7.6 ± 0.08^d^
MCMX, 0.0005%	42.69 ± 0.08^d^	51.67 ± 1.74^ab^	23.41 ± 0.08^bc^	4.16 ± 0.07^cd^	7.0 ± 0.08^b^
MCMX, 0.0007%	43.22 ± 0.08^e^	51.23 ± 1.74^ab^	23.26 ± 0.08^b^	4.22 ± 0.07^cd^	7.3 ± 0.08^c^
MCCX, 0.0002%	43.48 ± 0.08^e^	52.89 ± 1.74^ab^	23.51 ± 0.08^bc^	4.26 ± 0.07^cd^	6.4 ± 0.08^a^
MCCX, 0.0005%	43.91 ± 0.08^f^	53.56 ± 1.74^ab^	23.67 ± 0.08^cd^	4.39 ± 0.07^d^	7.2 ± 0.08^bc^
MCCX, 0.0007%	44.12 ± 0.08^f^	54.23 ± 1.74^b^	23.75 ± 0.08^d^	4.30 ± 0.07^d^	7.4 ± 0.08^cd^
MC without X	39.59 ± 0.08^a^	47.61 ± 1.74^a^	22.81 ± 0.08^a^	3.61 ± 0.07^a^	7.2 ± 0.08^bc^
CMC	40.16 ± 0.09^b^	49.89 ± 1.95^ab^	24.38 ± 0.08^e^	3.69 ± 0.09^ab^	7.6 ± 0.08^d^

MCMX = mozzarella cheese with microbial xanthan, MCCX = mozzarella cheese with commercial xanthan, MC = mozzarella cheese, X = xanthan, CMC = commercial mozzarella cheese, values equal percentage, TS = total solids, M = moisture, SNF = solids not fat.

For each storage period: overall mean values with different superscript letters differ significantly (*p < *0.05) in the same column, ±= standard error.

In Table 9, results showed that for total mesophilic count (TMC) (CFU/gm), there was a significant difference between all examined MC samples and commercial samples at 20 days of storage. For total staphylococci count (TSC) (CFU/gm), there was a significant difference between samples with MX at all concentrations and commercial samples at 10 days of storage, while there was a significant difference between all samples and commercial samples except MCMX 0.0007% at 30 days of storage. This indicates the reduced effect of previous types of added xanthan on staphylococci counts. For total fungi count (cells/gm), there was a significant difference between all the examined samples and commercial MC samples throughout the storage period.

**Table 9.** Microbiological statistical analytical results of the examined MC during different storage periods.

Treatment Parameter	1- MCMX, 0.0002%	2- MCMX, 0.0005%	3- MCMX, 0.0007%	4- MCCX, 0.0002%	5- MCCX, 0.0005%	6- MCCX, 0.0007%	7- MC without X	8- CMC
Storage period (zero-day)								
TMC, CFU/gm	1 × 10^7^ ± 3 × 10^6ab^	4.8 × 10^6^ ± 1.6 × 10^6a^	4 × 10^6^ ± 1.3 × 10^6a^	2.1 × 10^7^ ± 7 × 10^6c^	8.5 × 10^6^ ± 2.8 × 10^6ab^	4.2 × 10^6^ ± 1.4 × 10^6a^	9 × 10^7^ ± 3 × 10^7d^	1.8 × 10^7^ ± 6 × 10^6bc^
TSC, CFU/gm	2.3 × 10^4^ ± 7.6 × 10^3e^	4 × 10^3^ ± 1.3 × 10^3bc^	1.1 × 10^3^ ± 3.6 × 10^2a^	1.6 × 10^4^ ± 5.3 × 10^3d^	6 × 10^3^ ± 2 × 10^3c^	2 × 10^3^ ± 6.6 × 10^2ab^	1.6 × 10^4^ ± 5.3 × 10^3d^	3.5 × 10^4^ ± 1.1 × 10^4f^
Coliforms count (MPN/gm)	< 3^a^	< 3^a^	< 3^a^	< 3^a^	< 3^a^	< 3^a^	< 3^a^	9 ± 3.0^b^
TYMC (cells/gm)	3 × 10^3 ^± 1 × 10^3a^	1 × 10^3 ^± 3.3 × 10^2a^	< 10^a^	1 × 10^3^ ± 3.3 × 10^2 a^	< 10^a^	10^2^ ± 3.3 × 10^a^	2.5 × 10^3^ ± 8.3 × 10^2a^	2 × 10^5^ ± 6.6 × 10^4b^
Storage period (10 days)								
TMC	4.4 × 10^5^ ± 1.4 × 10^5a^	1.8 × 10^5^ ± 6 × 10^4 a^	5 × 10^4^ ± 1.6 × 10^4a^	8 × 10^6^ ± 2.6 × 10^6c^	7 × 10^5^ ± 2.3 × 10^5ab^	4.1 × 10^5^ ± 1.3 × 10^5a^	2 × 10^6^ ± 6.6 × 10^5b^	7.2 × 10^5^ ± 2.4 × 10^5ab^
TSC	5.4 × 10^4^ ± 1.8 × 10^4a^	5 × 10^3^ ± 1.6 × 10^3a^	1 × 10^3^ ± 3.3 × 10^2a^	2.5 × 10^5^ ± 8.3 × 10^4b^	2.3 × 10^5^ ± 7.6 × 10^4b^	2.7 × 10^5^ ± 9 × 10^4b^	4 × 10^3^ ± 1.3 × 10^3a^	2 × 10^5^ ± 6.6 × 10^4b^
Coliforms count	< 3^a^	< 3^a^	< 3^a^	< 3^a^	< 3^a^	< 3^a^	< 3^a^	2.1 × 10^2 ^± 7 × 10^ b^
TYMC	3 × 10^2^ ± 1 × 10^2a^	< 10^a^	< 10^a^	5 × 10^2^ ± 1.6 × 10^2a^	10^2^ ± 3.3 × 10^a^	10^2^ ± 3.3 × 10^a^	< 10^a^	2 × 10^4^ ± 6.6 × 10^3b^
Storage period (20 days)								
TMC	4.6 × 10^6^ ± 1.5 × 10^6e^	1 × 10^6^ ± 3.3 × 10^5b^	3.6 × 10^6^ ± 1.2 × 10^6d^	2.2 × 10^5^ ± 7.3 × 10^4ab^	1 × 10^4^ ± 3.3 × 10^3a^	2 × 10^4^ ± 6.6 × 10^3a^	1.5 × 10^6^ ± 5 × 10^5c^	1 × 10^8^ ± 3.3 × 10^7f^
TSC	6.7 × 10^5^ ± 2.2 × 10^5c^	2 × 10^3^ ± 6.6 × 10^2a^	3 × 10^3^ ± 1 × 10^3a^	2.4 × 10^4^ ± 8 × 10^3b^	1 × 10^3^ ± 3.3 × 10^2a^	1 × 10^3^ ± 3.3 × 10^2a^	2 × 10^3^ ± 6.6 × 10^2a^	4 × 10^3^ ± 1.3 × 10^3a^
Coliforms count	< 3^a^	< 3^a^	< 3^a^	< 3^a^	< 3^a^	< 3^a^	< 3^a^	4.3 × 10^2^ ± 1.4 × 10^2b^
TYMC	8 × 10^2^ ± 2.6 × 10^2a^	2 × 10^2^ ± 6.6 × 10^a^	< 10^a^	1.1 × 10^3^ ± 3.6 × 10^2a^	8 × 10^2^ ± 2.6 × 10^2a^	5 × 10^3^ ± 1.6 × 10^3a^	< 10^a^	4 × 10^5^ ± 1.3 × 10^5b^
Storage period (30 days)								
TMC	3.5 × 10^8^ ± 1.1 × 10^8d^	1 × 10^8^ ± 3.3 × 10^7ab^	8 × 10^8^ ± 2.6 × 10^8e^	8.6 × 10^7^ ± 2.8 × 10^7ab^	2.8 × 10^8^ ± 9.3 × 10^7cd^	6 × 10^6^ ± 2 × 10^6a^	5 × 10^7^ ± 1.6 × 10^7a^	2 × 10^8^ ± 6.6 × 10^7bc^
TSC	2 × 10^6^ ± 6.6 × 10^5b^	1.2 × 10^6^ ± 4 × 10^5ab^	6.6 × 10^6^ ± 2.2 × 10^6c^	4 × 10^5^ ± 1.3 × 10^5a^	1.3 × 10^5^ ± 4.3 × 10^4a^	6 × 10^4^ ± 2 × 10^4a^	4.5 × 10^5^ ± 1.5 × 10^5a^	6 × 10^6^ ± 2 × 10^6c^
Coliforms count	< 3^a^	< 3^a^	< 3^a^	< 3^a^	< 3^a^	< 3^a^	< 3^a^	1.1 × 10^5^ ± 3.6 × 10^4b^
TYMC	1.4 × 10^5^ ± 4.6 × 10^4ab^	2 × 10^5^ ± 6.6 × 10^4b^	1 × 10^3^ ± 3.3 × 10^2a^	3.6 × 10^4^ ± 1.2 × 10^4a^	1 × 10^3^ ± 3.3 × 10^2a^	5 × 10^3^ ± 1.6 × 10^3a^	5 × 10^3^ ± 1.6 × 10^3a^	8 × 10^5^ ± 2.6 × 10^5c^

Values equal mean ± standard error, overall mean values with different superscript letters differ significantly (*p* < 0.05) in the same row.

The significant indices of microbial quality are mesophilic, coliforms, fungi count, and specific microbe detection, as mentioned by Salazar-Llorente et al. [37]. The TSC appeared immediately after production, although the count (<10 CFU/ml) results after milk heat treatment, which indicates the availability of contamination during processing even with heat treatment and strict hygienic measures. Also, the high salt content (2.0%) and low water content of MC do not affect Staphylococci viability, which appeared obviously via a count increase until the end of the storage period in the refrigerator.* Staphylococcus aureus* could not be isolated from our produced MC and commercial samples due to the strict sanitary state in which the MC was processed, but the total staphylococci indicate a high possibility of *S. aureus* existence in the produced MC with elevated levels with storage, which may reach the toxigenic number (10^5 ^CFU/gm) [38]. For this reason, *S. aureus* was chosen as a model to prove the antibacterial influence of xanthan.

**Figure 3.**
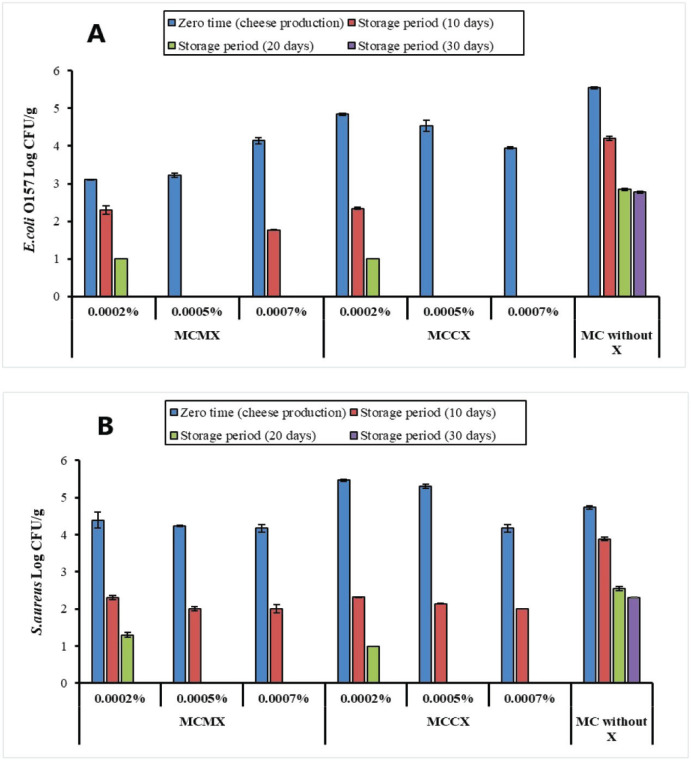
(A) Log reduction of inoculated* E. coli *O157 in the examined MC samples at different storage periods. (B) Log reduction of inoculated* S. aureus* in the examined MC samples at different storage periods. MCMX = mozzarella cheese with microbial xanthan, MCCX = mozzarella cheese with commercial xanthan, MC = mozzarella cheese, X = xanthan, illustrated values as mean, error bars indicate ± standard error, significant (*p* < 0.05) between all fortified samples and control samples throughout the storage period except MCMX 0.0002% at zero time for *S. aureus *only.

For the coliform count (MPN/gm), there was no significant difference (*p *> 0.05) between all samples containing xanthan and control samples throughout the storage period, and we failed to isolate *E. coli* in all the analyzed samples. The MC produced is free from coliforms. On the other hand, it was isolated at various levels from the commercial brands in the market. Also, the count was elevated during storage to a high number (1.1 × 10^5 ^MPN/gm). It is an indicator group for pathogenic Enterobacteriaceae [39], for which *E. coli* was chosen for the study. Bacteria caused 60.0% of MC recalls in the USA, of which coliforms alone participated in almost 33.0% [40]. The mean coliform count for MC containing xanthan samples was within the Egyptian standards (ES) limit (<10 cells/gm), while the commercial MC samples were not within the ES limit (<10 cells/gm) after 10, 20, and 30 days of storage. Increasing fungi counts from 10 days until the end of storage in commercial MC and lesser counts in other samples signal that fungi are less influenced by low moisture, salt, and refrigeration than bacteria. The fungi mean was higher than the limit (410 cells/gm) as mentioned by ES [41] for commercial MC samples throughout the storage period, while the mean fungi value in MCMX 0.0007% samples (<10 cells/gm) was within this limit. This indicates the reduced effect of MX on fungus counts.

Counts of TMC, staphylococci, and fungi were highly increased in the fourth week of storage in all samples, especially the commercial brand. For that, we advise storing MC in the refrigerator for no more than 20 days. MC wasted textural soundness and promoted unacceptable flavor after this period, proposing that incident microbes grew slowly at temperatures of 6°C–70°C. This signals that the state of MC (moisture range from 39.59% to 44.12%, 2.0% salt) is insufficient to prevent spoilage from microbial growth [11].

*Escherichia coli *and *S*. *aureus* were selected for survival trials because they exemplify Gram-negative and positive bacteria, respectively. The manner of growth of both selected bacteria after 30 days was analogous, with a reduction in counts for one month. Up to 2% NaCl supplemented in MC did not influence *E. coli* or *S. aureus* growth. *Escherichia coli *survived similarly after 30 days of storage in curd, suggesting that fecal contaminant bacteria may have similar survival patterns over longer storage times. The xanthan addition could significantly enhance *S. aureus* and *E. coli *O157 infection treatment by antimicrobials [11,42].

According to the* E. coli *O157 and *S. aureus* survival results (Figure 3), the* E. coli *O157 and *S. aureus* (log CFU/gm) could not be found in the examined samples with microbial and CX (0.0005% and 0.0007%) at 20 and 30 days of storage. The starter bacteria’s existence, reducing moisture, and xanthan addition may cumulatively inhibit low-fat soft cheese spoilage attributable to incident bacteria from the curd contact surfaces [36]. Bacterial survival was significantly (*p* < 0.05) reduced in the presence of xanthan at the end of the storage period by 2.77 and 2.3 logs on *E. coli *O157 and *S. aureus*, respectively. This log reduction is due to exaggerated acid output and nourishment reduction from xanthan [42].

Accordingly, reducing microbial presence during the processing of MC is critical for extending their period until consumption and safety enhancement. Also, this indicates the synergistic antimicrobial action of adding xanthan during the processing of MC. The survival trial from this investigation can be monitored as significant updated data for future research on MC safety. Xanthan utilization can enhance the organoleptic, meltability, chemical, and microbial qualities of MC. Supplemented xanthan in cheese will contribute to MC enhancement marketing. Hence, this work will contribute to improving standards that judge and control MC in the market. Excellent production practices and verifying the HACCP plan in the MC plant are essential to producing a high-safety MC [43]. The lab-produced xanthan needs broad extra investigations to realize its degree of antimicrobial effect in cheeses.

## Conclusion

The current study concluded that lab-produced xanthan (0.0007%) and CX (0.0005%) can be utilized to produce MC with excellent sensory parameters. The lab-produced xanthan (0.0005%) and CX (0.0002%) had a significant improvement in water content, which is a major aspect of enhancing MC performance throughout the storage period. Both types of xanthan with all concentrations had a significant improvement in MC fat percentage after 20 days of storage. The lab-produced xanthan (0.0002% and 0.0005%) and CX (0.0002%) can be added to reach the high meltability degree of MC at the end of storage. Both types and all concentrations of xanthan had a significant reducing influence on *E. coli *O157 and *S. aureus *counts in MC from 10 days until 30 days of storage. MC processors should be acquainted with this study’s information to take into account the addition of xanthan during the manufacturing steps of such a product.

## List of abbreviations

ATCC, American type culture collection; CFU, colony forming unit; CCD, central composite design; cm, centimeter; CP, centipoise; CX, commercial xanthan; ES: Egyptian standards; F, fat; gm, gram; h, hour; ISO, International Organization for Standardization; min, minute; MC, mozzarella cheese; MX, microbial xanthan; MPN, most probable number; OD, optical density; PCR, polymerase chain reaction; pH, potential of hydrogen; PB, Plackett–Burman; RPM, revolution per minute; RSM, response surface methodology; SNF, solids not fat; TP, total protein; TS, total solids; TMC, total mesophilic count; TSC, total staphylococci count; TYMC, total yeast and mold count; X, xanthan; YM, yeast malt.
